# Human Melanocyte-Derived Spheroids: A Precise Test System for Drug Screening and a Multicellular Unit for Tissue Engineering

**DOI:** 10.3389/fbioe.2020.00540

**Published:** 2020-06-04

**Authors:** Irina M. Zurina, Anastasiya A. Gorkun, Ekaterina V. Dzhussoeva, Tamara D. Kolokoltsova, Dmitriy D. Markov, Nastasia V. Kosheleva, Sergey G. Morozov, Irina N. Saburina

**Affiliations:** ^1^Laboratory of Cell Biology and Developmental Pathology, FSBSI Institute of General Pathology and Pathophysiology, Moscow, Russia; ^2^Department of Modern Biomaterials, Institute for Regenerative Medicine, Sechenov First Moscow State Medical University, Moscow, Russia; ^3^FSBEI FPE Russian Medical Academy of Continuous Professional Education of the Russian Ministry of Healthcare, Moscow, Russia; ^4^Institute of Molecular Genetics of the Russian Academy of Sciences, Moscow, Russia; ^5^Faculty of Biology, Lomonosov Moscow State University, Moscow, Russia

**Keywords:** melanocyte, melanogenesis, spheroid, 3D culture, drug screening, tissue engineering

## Abstract

Pigmentation is the result of melanin synthesis, which takes place in melanocytes, and its further distribution. A dysregulation in melanocytes' functionality can result in the loss of pigmentation, the appearance of pigment spots and melanoma development. Tissue engineering and the screening of new skin-lightening drugs require the development of simple and reproducible *in vitro* models with maintained functional activity. The aim of the study was to obtain and characterize spheroids from normal human melanocytes as a three-dimensional multicellular structure and as a test system for skin-lightening drug screening. Melanocytes are known to lose their ability to synthesize melanin in monolayer culture. When transferred under non-adhesive conditions in agarose multi-well plates, melanocytes aggregated and formed spheroids. As a result, the amount of melanin elevated almost two times within seven days. MelanoDerm™ (MatTek) skin equivalents were used as a comparison system. Cells in spheroids expressed transcription factors that regulate melanogenesis: MITF and Sox10, the marker of developed melanosomes—gp100, as well as tyrosinase (*TYR*)—the melanogenesis enzyme and melanocortin receptor 1 (*MC1R*)—the main receptor regulating melanin synthesis. Expression was maintained during 3D culturing. Thus, it can be stated that spheroids maintain melanocytes' functional activity compared to that in the multi-layered MelanoDerm™ skin equivalents. Culturing both spheroids and MelanoDerm™ for seven days in the presence of the skin-lightening agent fucoxanthin resulted in a more significant lowering of melanin levels in spheroids. Significant down-regulation of gp100, MITF, and Sox10 transcription factors, as well as 10-fold down-regulation of *TYR* expression, was observed in spheroids by day 7 in the presence of fucoxanthin, thus inhibiting the maturation of melanosomes and the synthesis of melanin. MelanoDerm™ samples were characterized by significant down-regulation of only MITF, Sox10 indicating that spheroids formed a more sensitive system allowed for quantitative assays. Collectively, these data illustrate that normal melanocytes can assemble themselves into spheroids—the viable structures that are able to accumulate melanin and maintain the initial functional activity of melanocytes. These spheroids can be used as a more affordable and easy-to-use test system than commercial skin equivalents for drug screening.

## Introduction

The pharmaceutical industry is one of the fastest-developing R&D systems. As long as new products are developed, discovery programs require more accurate and high-throughput *in vitro* systems for preclinical drug screening and testing. Currently, most *in vitro* studies are carried out using monolayer cultures of primary cells or immortalized cell lines. However, the efficacy of such approaches is now in doubt as 2D culture conditions do not fully reflect the complex 3D microenvironment (intercellular junctions, well-organized extracellular matrix, which cells have to make contact with, gradients of oxygen and nutrients) that surrounds cells *in vivo* (Fitzgerald et al., 2015).

Recently, 3D cell culture systems have been shown to model that microenvironment *in vitro*—they allow for maintaining cell morphology, viability, proliferation rate, and differentiation processes, as well as long-term culturing without disturbing the structural integrity (Antoni et al., 2015; Fitzgerald et al., 2015). Overall, this confirms that 3D cultures are more accurate and valuable in terms of predicting the clinical effects of tested drugs as compared to the 2D culture. There are now numerous technologies with which to obtain 3D cultures that can be divided into two categories: anchorage-independent and scaffold-based (Langhans, 2018). The first type of 3D culture is based on the ability of cells to self-organize in aggregates under non-adhesive conditions, including the hanging-drop technique, the use of low adhesion plates, and the magnetic cell levitation approach (Langhans, 2018). The use of multi-well non-adhesive plates is a simple way of obtaining a large number of spheroids of the same size and controlling their size by simply changing the cell concentrations (Koudan et al., 2017). In scaffold-based technologies, natural (ECM proteins) or synthetic scaffolds are used to provide mechanical support for cells, as well as to enable them to self-assemble in the preferred manner, similar to the native one (Langhans, 2018).

Numerous advantages of 3D cultures, mainly spheroids, have made them a widely used instrument for *in vitro* drug testing. The main area where spheroids are used is in the study of tumor cells and their resistance to different agents (Huang and Gao, 2018). That said, more researchers are now using 3D cultures for studying different pathologies and drug testing in cardiology (Figtree et al., 2017), neurology (Hartley and Brennand, 2017; Nzou et al., 2018), pulmonology (Surolia et al., 2017), orthopedics (Zigon-Branc et al., 2018), and endocrinology (Klaka et al., 2017; Ribeiro et al., 2018). New highly sensitive, yet easy-to-manipulate (and corresponding to the norms of bioethics) *in vitro* models are also required in cosmetic industries to study the efficacy and mechanisms of the effects of active drug substances or combined drugs.

The regulation of skin pigmentation, primarily skin lightening, is one of the most common processes in skin physiology, which is modified during cosmetological procedures. Active production of melanin, as well as its transfer from melanocytes to keratinocytes using special organelles—melanosomes—is one of the protective mechanisms of the skin against UV exposure. Melanin prevents the penetration of UV into the deeper layers of the skin and blocks the release of reactive oxygen species (ROS). Maintaining permanent skin pigmentation depends on a variety of sequential processes: migration of melanoblasts into tissue during embryogenesis, their viability and differentiation into melanocytes, the density of melanocytes in the skin, expression and functions of the enzymatic and structural components of melanosomes, synthesis of various types of melanin (eu- and pheomelanin), maturation and transportation of melanosomes in the dendritic processes of melanocytes and their transfer to keratinocytes, and, finally, the spread of melanin in the suprabasal layers of the skin (Yamaguchi and Hearing, 2007).

Therefore, to suppress hyperpigmentation and abnormal melanogenesis, the use of combined drugs that can affect different stages and levels of regulation of melanin synthesis is required. For example, fucoxanthin (algae pigment), which belongs to the carotenoid family and is used as a skin protector in cosmetology as a component in different drugs, has been shown to have multiple protective effects in various pathologies (Peng et al., 2011).

Currently, primary cultures of melanocytes and keratinocytes (Lei et al., 2002), the commercial tissue equivalents EpiSkin, MelanoDerm™ and others (Costin and Raabe, 2013; Meena and Mohandass, 2019) and tissue equivalents obtained from cells with induced pluripotency (iPSC) (Gledhill et al., 2015) are used to study *in vitro* the efficacy of drugs against hyperpigmentation. However, in this case, a simpler 3D model, namely, spheroids from melanocytes, could also be an effective tool for studying the mechanisms of anti-pigmentation drug efficacy. The 3D culturing of melanocytes in different systems has already been shown to reduce cell proliferation rates and increase their viability and functional activity (Lin et al., 2006; Lee et al., 2015; Hsiao and Young, 2019). The purpose of this study was to obtain and characterize spheroids from normal human melanocytes as a cellular module and as a test system for skin-lightening drug screening and to compare the efficacy of its use with MelanoDerm™ tissue equivalents.

## Materials and Methods

The study was conducted on the primary culture of human melanocytes (104-05N, CELL Applications, Inc.) and MelanoDerm™ tissue equivalents (MEL-300, MatTek Corporation).

### Cultivation of MelanoDerm™ Tissue Equivalents

A set of tissue equivalents was transported to the laboratory (at +4°C). The samples located in special holders were transferred to six-well culture plates in individual wells. Each sample was placed on a stand (MEL-STND, MatTek Corporation) on a section between the water and the air phases. Tissue equivalents were cultured in a complete growth medium provided by the company along with the samples (EPI-100-NMM-113, MatTek Corporation) under standard conditions (37°C, 5% CO_2_); the medium was replaced daily.

Fucoxanthin powder (3351-86-8, Anhui, China) was dissolved in saline buffer at a concentration of 500 μM (stock solution), then sterilized by filtration through a syringe filter (SLGP033RS, 0.22 μm, Millipore). For this study, the stock solution of fucoxanthin mixed with complete growth medium in a volume ratio of 1:10 (50 μM) was added to the experimental samples. The concentration of fucoxanthin was chosen based on the previously reported study on its protective effect on cell culture (Heo and Jeon, 2009) and our study of its cytotoxicity (data not published). An equivalent volume of saline buffer (1:10) was added to the control samples.

### 2D Cultivation of Human Melanocytes

The culture of human melanocytes was transported to the laboratory (at −20°C), and the cells were thawed in a water bath at 37°C. Cells were resuspended in a complete growth medium for melanocytes (135–500, CELL Applications, Inc.) and placed on Petri dishes (35 mm) at a density of 10^4^ cells/cm^2^. When the melanocytes reached a confluent state, the culture was passaged with Versene solution (R080p, PanEco) and 0.25% trypsin solution (R036p, PanEco). The full growth medium was replaced every 2 days. For further experimental studies, the culture of melanocytes at passage 4 was used to obtain spheroids.

### 3D Cultivation of Human Melanocytes

Human melanocytes were cultured under 3D non-adhesive conditions with and without the addition of the drug, to obtain three-dimensional spheroids and to study the effect of fucoxanthin on melanin accumulation by melanocytes under these conditions, which are closer to the native tissue compared to monolayer culture. Agarose plates were obtained by polymerizing 2% agarose type I (A6013, Sigma) in special plastic forms (12–256, 3D Petri Dishes, Microtissue) and were then placed in 12-well culture plates. Cell suspension was obtained from the monolayer melanocyte culture at passage 4, resuspended in the full growth medium, and placed in agarose plates at a concentration of 1 × 10^3^ cells per micro-well. 50μM of fucoxanthin was added to experimental spheroids. In the control group, the same volume of sodium chloride (NaCl) buffer was added to the growth medium. The dynamics of spheroids' formation in agarose plates were monitored by the Cell-IQ live time-lapse system (CM Technologies, Finland) with photo registration every 20 min. The resulting spheroids were collected on Days 1, 3, and 7 for melanin concentration measurements and real-time PCR, and at Days 3 and 7 for immunocytochemical analysis.

### Photometric Analysis of Melanin Concentration in MelanoDerm™ Tissue Equivalents and Spheroids From Human Melanocytes

Prior to photometric analysis, the samples of MelanoDerm™ (3 pieces of tissue equivalent per time point in experimental and control groups) and spheroids (256 spheroids per time point in experimental and control groups) were washed three times from the residues of the medium in PBS (pH = 7.4), after which they were stored at −20°C. To extract melanin, the samples were thawed and dried, and then 250 μl of Solvable solution (6NE9100, PerkinElmer) was added to each sample. Next, the samples were incubated for 18 h in a water bath at +60°C. After incubation, the samples were thoroughly mixed in a vortex mixer, undissolved particles were precipitated by centrifugation (5 min, 13,000 g), 100 μl of the supernatant was placed into a 96-well plate, and samples' optical densities were measured at a wavelength of 490 nm on a Multiscan GO plate photometer (Thermo Scientific, USA). A calibration curve was obtained by analyzing standard solutions with a known concentration of melanin prepared from dry matter (M863, Sigma-Aldrich). Measurements were performed in triplicate.

### Fixation of MelanoDerm™ Tissue Equivalents and Spheroids From Human Melanocytes

For immunocytochemical analysis of melanocyte monolayer culture, cells were seeded on the cover glass. Before fixing the 3D culture, the spheroids were collected in the tube and centrifuged (1 min, 100 g). The resulting pellet was washed three times in phosphate-saline buffer solution (PBS, pH = 7.4). Cover glasses seeded with melanocytes and spheroids were then fixed in a 4% solution of paraformaldehyde (20 min, +4°C). MelanoDerm™ tissue equivalents were also washed three times with PBS from the remnants of the culture medium (5 min for each wash, pH = 7.4). The material was fixed in a 4% solution of paraformaldehyde (one day, +4°C).

### Immunocytochemistry

After fixation, the MelanoDerm™ tissue equivalents samples were dehydrated in Isoprep histological processing solution (06-002/S, Biovitrum) and embedded in paraffin (01-007/1, Biovitrum). Next, a series of 9 μm sections was made on the microtome; the sections were placed on glass slides. Prior to immunocytochemical staining, the paraffin was removed from the sections in three changes of xylene (5 min each) and a series of alcohols with descending concentrations (1 min each) to distilled water. Next, the antigens were unmasked in citrate buffer (pH = 6.0; 06-014, Biovitrum) for 3 min at +100°C under pressure. Cover glasses and spheroids were washed three times with PBS (5 min for each wash, pH = 7.4) from the remnants of PFA. All the following staining procedures were the same for 2D culture, spheroids, and tissue equivalent samples. Melanocytes in monolayer culture were incubated with primary antibodies against MEL5 (917801, BioLegend, 1:100). After preliminary preparations, sections and spheroids were incubated with primary antibodies against gp 100 (ab137078, Abcam, 1:100), Sox10 (ab155279, Abcam, 1:500), and MITF (ab122982, Abcam, 1:300). The result was visualized using secondary antibodies conjugated with the fluorochromes FITC (Em = 525 nm) and DyLight594 (Em = 617 nm). The nuclei were stained with a bisbenzimide fluorescent dye, i.e., Hoechst 33258 (0.002 mg/ml, 10 min, 25°C). After staining, the excess dye was removed in three changes of PBS (pH = 7.4). The resulting preparations were placed in Vitrogel mounting medium (12-001, Biovitrum) and analyzed in visible and ultraviolet light ranges using the Olympus Fluoview FV10 laser scanning confocal microscope (Olympus, Japan).

The obtained images of MelanoDerm™ sections and spheroids from melanocytes were analyzed for mean fluorescence intensity. Images were imported into ImageJ software and converted to a 16-bit format. The parameters “area integrated intensity” and “mean gray value” were measured in the area limited by the threshold. The corrected total cell fluorescence (CTCF) was calculated using the formula: CTCF = integrated density—(area of selected cell × mean fluorescence of background readings). Three images were measured for each marker and time point.

### Analysis of Gene Expression by Real-Time Polymerase Chain Reaction

To perform real-time PCR, three pieces of MelanoDerm™ and 256 spheroids from melanocytes were used for each time point in experimental and control groups. Total RNA was isolated using a standard Trizol method (TRIReagent, Sigma, USA). The RNA was treated with Type I DNase (Fermentas, Germany) to remove any DNA contamination, and precipitated in 4M LiCl. The concentration of the obtained RNA was measured using a Nanodrop 8000 spectrophotometer (Thermo Scientific, USA); 2 μg of total RNA were used to synthesize cDNA. cDNA synthesis was performed using M-MLV reverse transcriptase (Evrogen, Russia) and random hexanucleotides (Sileks, Russia). The obtained samples were used to analyze the expression of the *MC1R* gene and the *TYR* gene. The sequences of the used primers are presented in Table 1. The analysis was performed on an automatic 7500 Real-Time PCR System amplifier (Applied Biosystems, USA) using a qPCRmix-HSSYBR + ROX mixture (Evrogen, Russia). The relative expression of *MC1R* and *TYR* genes was measured using the ΔΔCt method, normalized to *HsTBP* (Bookout et al., 2006).

**Table 1:** Primers used in this research.

**Primers**	**Primer sequence (5^**′**^-3^**′**^)**	**Length**	**Tm**°**C**	**Primer description**
MC1R_For	GTGGTCTTCTTCCTGGCTATGC	22 bp	62.3	*MC1R*
MC1R_Rev	GGATGGTGAGGGTGACAGCG	20 bp	63.0	
TYR_For	TTCAAGAAGTTTATCCAGAAGCC	23 bp	57.7	*TYR*
TYR_Rev	CTTAATGTAGTCTTGAAAAGAGTC	24 bp	53.8	
HsTBP_For	CATGACTCCCGGAATCCCTATCTTT	25 bp	63.1	*HsTBP*
HsTBP_Rev	TGTTGCTGCTGCTGCCTTTGTT	22 bp	63.7	

### Statistics

Statistical analysis was performed, and graphs were created using the Prism 8.0 GraphPad software package. Using the Shapiro-Wilk, hypotheses about the normality of the distributions of the studied parameters, and the Pearson χ2 test for the evaluation of fucoxanthin influence were tested. Both two-way ANOVA assay and multiple *t*-test were used consecutively to confirm the significant difference of data. The *p*-value was adjusted using the two-stage linear step-up procedure of Benjamini, Krieger, and Yekutieli, with Q = 5%. All the experiments were reproduced in triplicate; data are reported as the means ± SD of at least three experiments.

## Results

### Study of Melanin Accumulation in 3D Cultures—Spheroids From Melanocytes and MelanoDerm™ Tissue Equivalent

**Figure 1:**
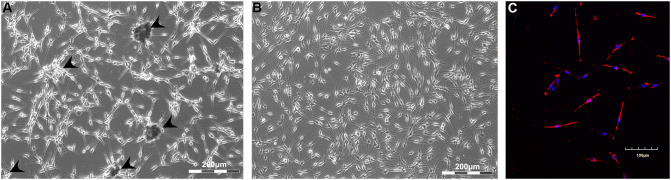
Monolayer culture of human melanocytes at Passages 1 **(A)** and 4 **(B)**. Cells were shown to express Mel5—the marker of melanocytes **(C)**. Arrowheads indicate the sites of aggregates formation. **(A,B)**—Light phase-contrast microscopy; **(C)**—laser scanning confocal microscopy.

Primary human melanocytes (CELL Applications) were first maintained in monolayer culture. During the first passages, melanocytes mostly had spindle-like morphology, with a tendency to the formation of dark aggregates (Figure 1A). By the fourth passage, cell morphology slightly changed—cells had more dendrites, while no aggregates were observed in the culture (Figure 1B). Cells were characterized by the positive staining against melanocyte-specific marker MEL5, also known as a tyrosinase-related protein-1 that is involved in melanin synthesis (Figure 1C).

**Figure 2:**
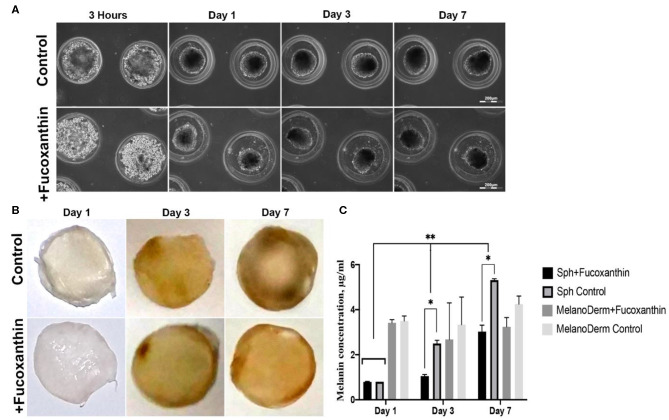
Pigmentation of treated and untreated spheroids and skin equivalents. **(A)** Dynamics of the spheroid formation from a suspension of melanocytes under non-adhesive 3D conditions in a standard growth medium (control) and with the addition of fucoxanthin (experimental group). Phase-contrast live time-lapse microscopy (Cell-IQ, CM Technologies, Finland). **(B)** Pigment accumulation in MelanoDerm™ tissue equivalents at Days 1, 3, and 7 in the control group and the experimental group with the addition of fucoxanthin. All samples had the standard size−9 mm. Photos of samples dried before photometric analysis of melanin content. **(C)** Dynamics of melanin accumulation in melanocyte spheroids and MelanoDerm™ tissue equivalents in the presence of the lightening agent fucoxanthin (experimental group) and with a solution of sodium chloride (control group). *p < 0.05 for two-way ANOVA assay and multiple *t*-test. *, **p < 0.05 (*within one time-point, **between different time-points).

The melanocytes at the fourth passage were placed in non-adhesive agarose multi-well plates to obtain 3D spheroid culture. Using the method of live time-lapse microscopy, we observed the dynamics of spheroid formation in the control culture and in the presence of fucoxanthin (Figure 2A). In the process of culturing cells under 3D conditions, general patterns of spheroid formation were observed—initial compaction took place during the first 24 h of cultivation and lasted up to 7 days. Moreover, by Day 7 both control and experimental spheroids became darker (Figure 2A), but the difference between groups could not be adequately assessed visually because spheroids were dense and not transparent.

MelanoDerm™ tissue equivalents are artificially created tissue samples consisting of layers of keratinocytes and melanocytes cultured on a porous membrane. Throughout the cultivation period, the samples accumulated melanin by increasing the number of melanocytes and autoregulating the intensity of pigment synthesis. The visual analysis showed that while on the first and third days the dynamics of samples darkening did not differ significantly, by Day 7, the experimental skin equivalents cultured in the presence of fucoxanthin were lighter compared to the control group (Figure 2B).

To support the visual data on melanin accumulation in these two types of 3D culture containing melanocytes, the spectrophotometric analysis of pigment concentration was performed. The obtained data confirmed the visual assessment of the intensity of melanin synthesis in control and experimental spheroids. As shown in Figure 2C, on Day 1 in 3D culture, the average concentration of melanin was similar in both groups, but, on Days 3 and 7, melanin synthesis by spheroids in the presence of fucoxanthin was significantly lower than in the control group, as confirmed by statistical analysis.

The data from the visual observations of melanin accumulation in MelanoDerm™ were also confirmed using photometry (Figure 2C). The obtained results showed that melanin content in control and experimental groups was the same on the first day of cultivation, while, on Day 3, a decrease in the melanin content was observed in the experimental group relative to the control group. By Day 7, the final concentration of melanin in the experimental samples after cultivation with fucoxanthin was significantly lower than in the control group, but it slightly increased compared to Day 3 in the experimental group. However, no statistically significant difference was observed between the groups.

### Immunocytochemical Analysis of 3D Cultures

To assess the effect of fucoxanthin on the expression of the key factors of melanogenesis gp 100, MITF and Sox10, immunocytochemical analysis of the MelanoDerm™ tissue equivalent sections and spheroids from melanocytes was performed.

**Figure 3:**
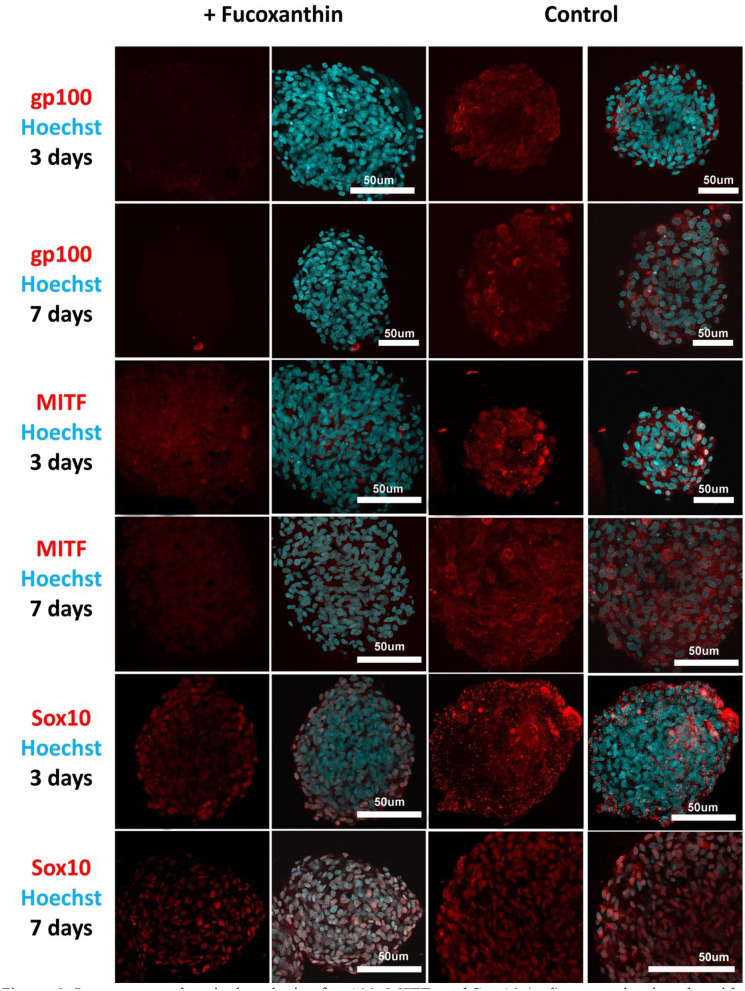
Immunocytochemical analysis of gp100, MITF, and Sox10 (red) expression in spheroids from human melanocytes in the control and experimental groups on Days 3 and 7 in 3D culture. The nuclei are stained with Hoechst 33258 (blue). Laser-scanning confocal microscopy.

Visual comparison of stained spheroids revealed no difference in the expression of gp100 on Day 3. However, there was up-regulation on Day 7 in the control group as compared to the fucoxanthin-present group (Figure 3). On the other hand, MITF expression was lower on day 3 and showed up-regulation on the day 7th in the spheroid control group as compared to the fucoxanthin present spheroid group (Figure 3). The expression of the Sox10 was higher on the 3rd day as well as 7th day in the spheroid control group as compared to the fucoxanthin-present spheroid group (Figure 3). We also analyzed the images using ImageJ for digital analysis (Figure 5). The data obtained were consistent with the visual analysis.

**Figure 4:**
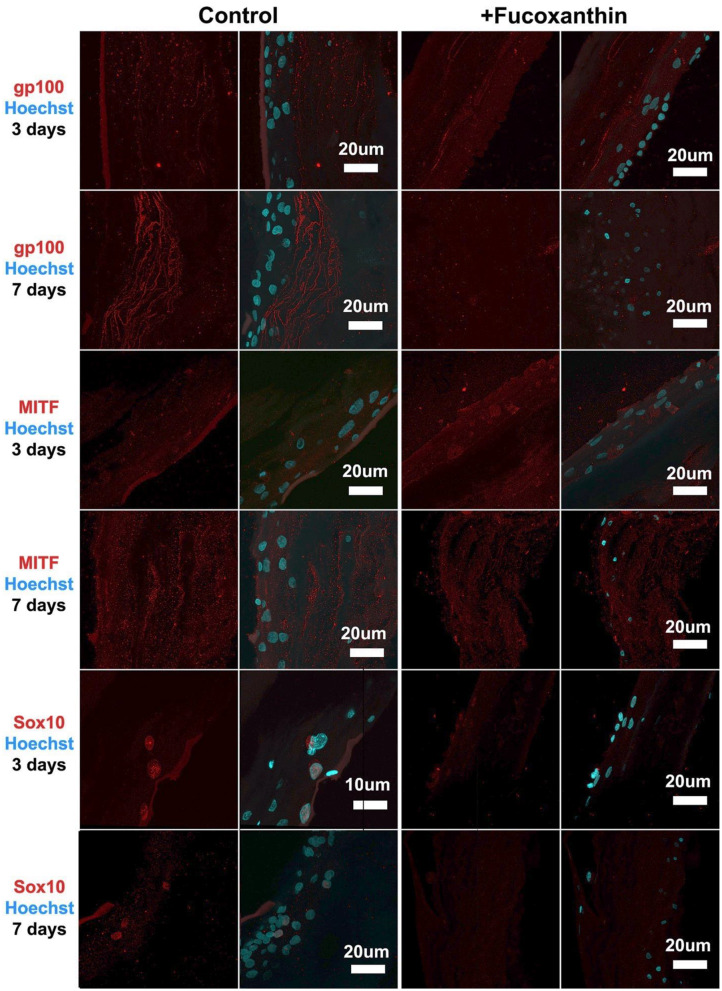
Immunocytochemical analysis of gp100, MITF, and Sox10 (red) expression on sections of MelanoDerm™ tissue equivalents in the control and experimental groups on Days 3 and 7 of cultivation. The nuclei are stained with Hoechst 33258 (blue). Laser-scanning confocal microscopy.

**Figure 5:**
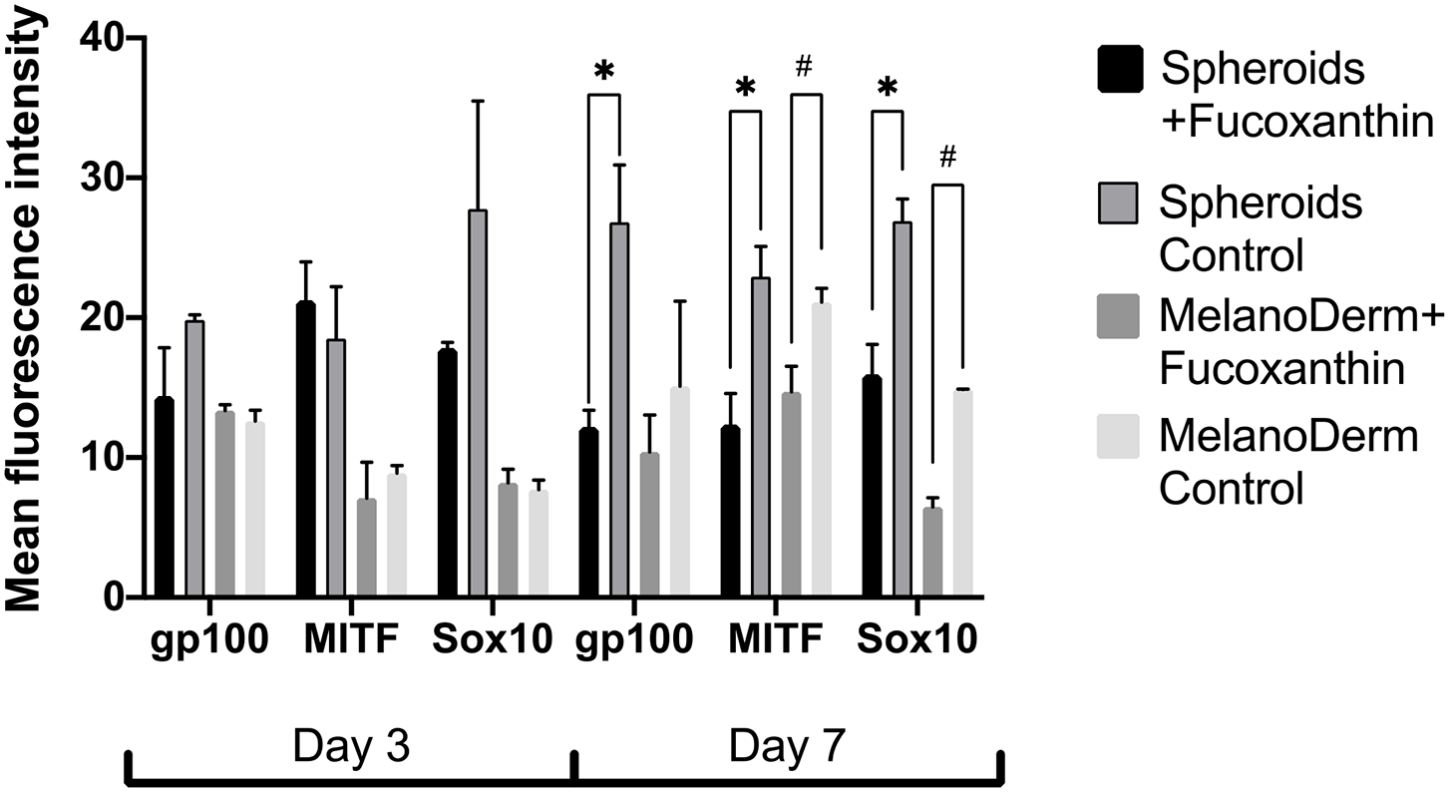
The analysis of mean fluorescence intensity on the immunocytochemical staining images of spheroids and MelanoDerm™ sections in the experimental (in the presence of fucoxanthin) and control groups presenting an expression of gp100, MITF, and Sox10 on Days 3 and 7 in 3D culture. *^#^*p* < 0.05 for two-way ANOVA assay and multiple *t*-test.

For the MelanoDerm™ group, it was shown that the expression level of gp100, which is responsible for the maturation of melanosomes, was increased in the MelanoDerm™ control group compared to the MelanoDerm™ experimental group on Day 7 of cultivation (Figure 4). On Days 3 and 7, there was a slight increase in the expression level of MITF in the presence of the drug, as well as in the MelanoDerm™ control group with a high level of expression of this transcription factor (Figure 4). Sox10 on Days 3 and 7 of cultivation was expressed in cell nuclei in the control MelanoDerm™ samples and practically not present in the experimental samples (Figure 4). These observations were supported by a quantitative analysis of the mean fluorescence intensity of the obtained images (Figure 5).

### Real-Time PCR Analysis of Melanogenesis Factor Expression in MelanoDerm™ Tissue Equivalents and Spheroids From Human Melanocytes

**Figure 6:**
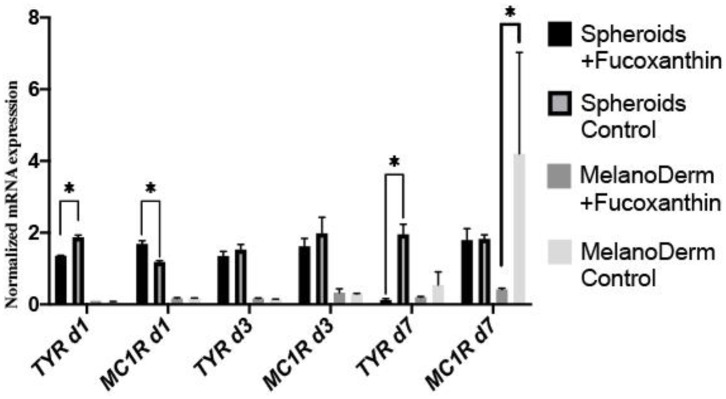
Real-time PCR analysis of *TYR* and *MC1R* expression in MelanoDerm™ tissue equivalents and spheroids from human melanocytes on Days 1, 3, and 7 in the presence of fucoxanthin and in the control group with growth medium. *p < 0.05 for two-way ANOVA assay and multiple *t*-test.

Real-time PCR revealed the effect of fucoxanthin on the expression of tyrosinase (*TYR*)—one of the melanogenesis enzymes. That is, it was down-regulated by Day 7 in both spheroids and skin equivalents, compared to the corresponding control groups, but, in the case of spheroids, the difference between the control and experimental groups was significant on Days 1 and 7 (Figure 6). The gene expression of the main receptor regulating melanin synthesis, i.e., melanocortin receptor 1 (*MC1R*), was affected by fucoxanthin only in MelanoDerm™ culture, which was confirmed by statistical analysis (Figure 6).

## Discussion

The expanding ozone holes in the atmosphere lead to the growing danger of UV skin damage, making the pharmaceutical industry increase the production of new types of sun protection (Smit et al., 2009; Zastrow et al., 2017), which requires the new systems for testing them *in vitro*. At present, the mainly used test-systems are primary cultures of melanocytes and keratinocytes (Lei et al., 2002; Lee et al., 2020), melanoma cell lines (Kim et al., 2017; Lee et al., 2020), the commercial tissue equivalents (EpiSkin™, MelanoDerm™, and others) (Costin and Raabe, 2013; Kim et al., 2017; Meena and Mohandass, 2019; Lee et al., 2020), and tissue equivalents obtained from cells with induced pluripotency (iPSC) (Gledhill et al., 2015). These approaches have some limitations, including non-physiological conditions (for 2D cultures), difficulties in analysis, and expensiveness (for commercial tissue equivalents). The current study was aimed to assess the ability of spheroids from normal human melanocytes to maintain their functional activity (melanin synthesis) and to react to the addition of hypopigmentation agent fucoxanthin. Additionally, we compared spheroids' properties to 3D MelanoDerm™ skin tissue equivalents, which we considered as the closest available alternative.

Melanocytes had cell-specific dendritic morphology and expressed specific marker Mel5 at passage 4 in the standard monolayer system (Figure 1). However, cells were not able to accumulate melanin *in vitro*, which was corresponded by numerous studies (Chung et al., 2019). At the first passages, cells demonstrated a tendency to the spontaneous formation of dark aggregates attached to the surface, which has been previously reported only on specific culture surface (Lin et al., 2005). This effect was no longer observed at the further passages. When transferred in non-adhesive agarose microplates, melanocytes formed pigmented spheroids that accumulated melanin during cultivation. The same tendency was observed in commercial skin equivalent MelanoDerm™ (Figure 2).

In our study, we used the well-known skin-lightening compound fucoxanthin, which targets melanocytes, to validate the capability of both spheroids from melanocyte and skin equivalents of the tissue-specific reaction (the regulation of melanin synthesis). Fucoxanthin is a naturally occurring brown- or orange-colored pigment that belongs to the class of non-provitamin A carotenoids present in the chloroplasts of brown seaweeds (Martin, 2015). Fucoxanthin is well-known as the compound that affects different cellular pathways providing antioxidative effects (Martin, 2015) and hypopigmentation (Shimoda et al., 2010). Both spheroids from melanocyte and MelanoDerm™ showed a decrease of melanin synthesis in the presence of fucoxanthin, but that difference between control and experimental groups was significant only for spheroids (Figure 2), probably, because they contained only melanocytes.

**Figure 7:**
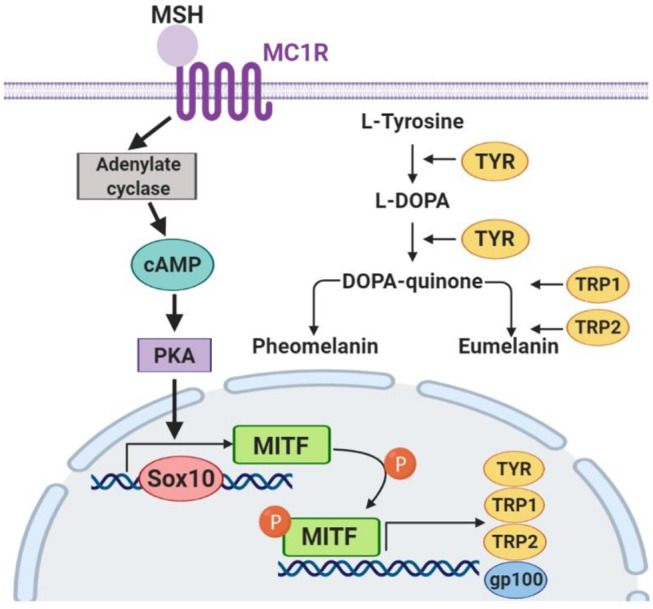
The key components of the melanogenesis signaling pathway.

To confirm the mechanisms of fucoxanthin physiological influence, we studied the expression of key factors that regulate melanogenesis. Since the synthesis of melanin is a complex multi-stage process (Figure 7) controlled by both endogenous and exogenous factors, and fucoxanthin, as well as other carotenoids, is a multitargeting agent (Shimoda et al., 2012), we have analyzed elements from different levels of this melanogenesis signaling pathway (Figure 7).

The skin pigment synthesis starts with the activation of melanocortin 1 receptor (MC1R) by its agonists—α-melanocyte-stimulating hormone (αMSH) and adrenocorticotropic hormone (ACTH) (Slominski et al., 2004). This activation stimulates the cAMP/PKA cascade leading to the increased expression of the master regulator of melanocyte development, i.e., microphthalmia-associated transcription factor (MITF) (Yamaguchi and Hearing, 2007). MITF, in its turn, activates the expression of the *TYR* gene (tyrosinase), *PMEL17* (or gp100—premelanosome protein), and Bcl-2 (anti-apoptotic factor), which leads to an increased synthesis of melanin (Lin and Fisher, 2007). The transcription factors Sox10 and PAX3, in synergistic terms, directly regulate the expression of MITF (Bondurand et al., 2000; Hou et al., 2006). In addition, the intensity of melanin synthesis in melanocytes directly depends on the amount and activity of the tyrosinase enzyme in the cells, which catalyzes the first two stages of melanin formation: hydroxylation of L-tyrosine into L-dihydroxyphenylalanine (L-DOPA) and subsequent oxidation of L-DOPA into L-dopaquinone.

As for the anti-melanogenic effect of fucoxanthin, it is shown to reduce TYR activity, the expression of MC1R, TYR-dependent receptor 1, cyclooxygenase 2 (COX-2), prostaglandin receptor 1 (EP1), which also indicates its anti-inflammatory properties (Shimoda et al., 2010). A general overview shows that fucoxanthin inhibits TYR and melanin production *in vitro* in B16 melanoma cells and *in vivo* in guinea-pigs and mice skin. It also suppressed PGE2, MSH, TRP1, and melanogenic stimulant receptors—EP1and MC1R *in vivo* (Azam et al., 2017). Based on these findings, we selected to assay the expression of *MC1R* and *TYR* mRNA (Figure 6).

Our study demonstrated significant down-regulating of *TYR* gene expression on days 1 and 7 for melanocyte spheroids and reduction of *MC1R* gene expression on day 7—for MelanoDerm™. Thus, melanocyte spheroids reproduced results described previously *in vitro* and *in vivo*, and MelanoDerm™ system showed results matched with *in vivo* studies. However, neither system showed both results.

Fucoxanthin is also interacting with other signaling pathways such as NF-κB and WNT/β-catenin pathways (Martin, 2015), which are also involved in melanogenesis (D'Mello et al., 2016). Using this fact, we have also analyzed other crucial factors of melanogenesis—the cellular expression of transcription factors Sox10 and MITF, and the expression of the melanosome maturation regulator—gp100/PMEL17 (Figures 3, 4). Melanocyte spheroids and skin equivalent MelanoDerm™ equally showed the significant reduction of expression of the transcription factors MITF and Sox10 in the presence of fucoxanthin (Figure 5). However, only spheroid model has firstly revealed that the application of fucoxanthin can inhibit melanosome formation by significantly decreasing gp100 synthesis. Thus, in general, the spheroid model showed higher responsiveness to fucoxanthin, as confirmed by statistical analysis.

The obtained results indicate that, in the case of studies of agents that potentially affect pigment synthesis and physiology of melanocytes, spheroids can be used as a more affordable and reproducible drug-testing system. The preservation of native-like cell morphology and physiology in spheroids makes it possible to conduct a study on isolated cells instead of using expensive tissue equivalents consisting of different types of cells and requiring special conditions for culturing.

## Conclusions

Collectively, these data illustrate that the 3D culture system can maintain the melanin accumulation *in vitro* compared to the MelanoDerm™ skin equivalents. Moreover, the obtained spheroids provided a sensitive response to the compound addition, recapitulated the *in vitro* and *in vivo-*like response (reduction of MITF, Sox10, and TYR expression), and showed new data (down-regulation of gp100 expression) that has not been previously reported using MelanoDerm™ or other skin tissue equivalents. Thus, melanocyte spheroids can be used as a convenient and affordable test system for screening drugs targeting melanocytes and potentially as building blocks for the tissue engineering of skin equivalents.

## Data Availability Statement

All datasets generated for this study are included in the article/supplementary material.

## Author Contributions

IZ, AG, and IS contributed to the conception and the design of the study. IZ wrote the first draft of the manuscript and prepared the submitted version together with AG. IZ and ED cultivated Skin Equivalents (MelanoDerm™) and spheroids, collected samples. ED and TK conducted all 2D cell culture works. AG accomplished the immunocytochemical analysis of all samples. DM performed the molecular assay and the photometric assay of all samples. NK provided statistical analysis and created the graphs. SM and IS supervised the study and edited the manuscript. All authors contributed to manuscript revision, read, and approved the submitted version.

## Conflict of Interest

The authors declare that the research was conducted in the absence of any commercial or financial relationships that could be construed as a potential conflict of interest.
